# Characterization of the ABA Receptor VlPYL1 That Regulates Anthocyanin Accumulation in Grape Berry Skin

**DOI:** 10.3389/fpls.2018.00592

**Published:** 2018-05-18

**Authors:** Zhen Gao, Qin Li, Jing Li, Yujin Chen, Meng Luo, Hui Li, Jiyuan Wang, Yusen Wu, Shuyan Duan, Lei Wang, Shiren Song, Wenping Xu, Caixi Zhang, Shiping Wang, Chao Ma

**Affiliations:** ^1^Department of Plant Science, School of Agriculture and Biology, Shanghai Jiao Tong University, Shanghai, China; ^2^Institute of Agro-Food Science and Technology, Key Laboratory of Agro-Products Processing Technology of Shandong, Shandong Academy of Agricultural Sciences, Jinan, China

**Keywords:** grape, anthocyanin, PYL1, ABA sensitivity, gene over-expression

## Abstract

ABA plays a crucial role in controlling several ripening-associated processes in grape berries. The soluble proteins named as PYR (pyrabactin resistant)/PYL (PYR-like)/RCAR (regulatory component of ABA receptor) family have been characterized as ABA receptors. Here, the function of a grape PYL1 encoding gene involved in the response to ABA was verified through heterologous expression. The expression level of *VlPYL1* was highest in grape leaf and fruit tissues of the cultivar Kyoho, and the expression of *VlPYL1* was increased during fruit development and showed a reduction in ripe berries. Over-expression of *VlPYL1* enhances ABA sensitivity in Arabidopsis. Using the transient overexpression technique, the *VlPYL1* gene was over-expressed in grape berries. Up-regulation of the *VlPYL1* gene not only promoted anthocyanin accumulation but also induced a set of ABA-responsive gene transcripts, including *ABF2* and *BG3*. Although tobacco rattle virus (TRV)-induced gene silencing (VIGS) was not successfully applied in the “Kyoho” grape, the application of the transient overexpression technique in grape fruit could be used as a novel tool for studying grape fruit development.

## Introduction

Fruit development and ripening are complex plant processes. Fruits are classified into climacteric and non-climacteric depending on respiration and ethylene production during maturation and senescence (Giovannoni, 2001). From extensive studies on climacteric fruits, such as the tomato, banana, peach, apple, and melon fruit, ripening has been shown to be marked by a respiratory burst linked to the production of ethylene, which plays a pivotal role in the regulation of climacteric fruit ripening (Alexander and Grierson, 2002; Adams-Phillips et al., 2004; Gupta et al., 2006; Pech et al., 2008; Cherian et al., 2014). Contrastingly, in non-climacteric fruits, such as the strawberry, grape, orange, cucumber, bilberry, and blueberry, a respiratory burst does not occur, and ethylene may have a less central impact on ripening, while ABA appears to have a pivotal regulatory role in the process (Ji et al., 2012; Zifkin et al., 2012; Karppinen et al., 2013; Wang et al., 2013; Cherian et al., 2014; Jia et al., 2016).

Grapevines are cultivated world-wide and are an economically valuable fruit crop. The development and ripening process of grape berries have their own characteristic with a double sigmoid growth pattern, which is resulting from two rapid stages of growth separated by a lag phase of slow or no growth (Coombe and McCarthy, 2000; Xie et al., 2009). Berry acidity increases during the initial stage of rapid berry growth and declines sharply after the lag phase, while anthocyanin and sugar contents increase rapidly (Xie et al., 2009). Comprehensive studies have shown that ABA is an important hormone in the inception and color development stage of grape berries ripening (Ferrara et al., 2015; Fortes et al., 2015; Jia et al., 2017; Pilati et al., 2017). A strong rise in berry ABA content corresponds with the increase in total anthocyanin content that is recorded around the *véraison* and during the initial stages of ripening (Wheeler et al., 2009; Lacampagne et al., 2010; Sun et al., 2010; Wang et al., 2016). Anthocyanins keep increasing throughout the ripening phase, while ABA levels simultaneously start to decrease (Wheeler et al., 2009; Wang et al., 2016), implying that ABA triggers but does not necessarily maintain color acquisition (Kuhn et al., 2014). Additionally, treatments by ABA cause an advance in red color development including Olympia (Hiratsuka et al., 2001a), Kyoho (Ban et al., 2003), Crimson Seedless (Cantin et al., 2007; Ferrara et al., 2013), Cabernet Sauvignon (Wheeler et al., 2009), Alicante Bouschet (Castellarin et al., 2011) and Fujiminori (Jia et al., 2017).

Cells need to sense ABA and then transduceit to the cellular components for ABA function (Nakashima and Yamaguchi-Shinozaki, 2013). In 2009, independent groups simultaneously discovered the structural and functional mechanisms of ABA sensing by cytosolic PYRABACTIN RESISTANCE1(PYR1)/PYR1-like (PYL)/REGULATORY COM PONENTS OF ABA RECEPTORS (RCAR) receptor proteins (Ma et al., 2009; Park et al., 2009). PYR/PYL/RCAR proteins bind to ABA and thus inhibit the activity of PP2C proteins (Ma et al., 2009; Park et al., 2009), leading to the derepression of SnRK2 protein kinases and the activation of ABA-responsive binding factors (ABFs), which are thought to be necessary to promote ABA-induced gene expression (Kobayashi Y. et al., 2005; Umezawa et al., 2009).

The PYR/PYL/RCAR family contains 14 members (PYR1 and PYL1-13) in Arabidopsis, 12 members in rice (He et al., 2014) and 14 members in tomatoes (Gonzalez-Guzman et al., 2014). In Arabidopsis, all of the 14 members can activate ABA-responsive gene expression (Fujii and Zhu, 2009; Fuchs et al., 2014). Many studies have shown that perception of ABA by the PYR/PYL/RCARs plays a critical role in the regulation of seed germination, seedling establishment, vegetative and reproductive growth, stomata closure, and transcriptional responses to the hormone (Nakashima and Yamaguchi-Shinozaki, 2013). As ABA receptors, PYR/PYL/RCAR will be of great value in increasing ABA sensitivity (Zhang et al., 2013b).

One ABA receptor from grape, *VvPYL1* can bind ABA and then inhibit the ABI1 phosphatase activity (Li et al., 2012). Eight PYR/PYL/RCARs, four of which have been identified and induced by ABA in the leaves of grapevines (Boneh et al., 2012a,b). Also, high levels of *VvPYL1* (*VvRCAR7*) expression were induced in grape leaves by drought, salt and cold stress (Boneh et al., 2012b). Recently, the ABA receptor PYR1 was cloned from the cultivar “Fujiminori,” whose expression levels were low in the early stage of fruit development and increased rapidly in the former stage of the grape berry, being PYR1 expression pattern consistent with ABA content changes (Jia et al., 2017). However, solid evidence has not been provided to verify the function of *VvPYL1* during the ripening process of grape fruits.

In this study, the data on the ripening-related expression of *PYL1* in the grape cultivar “Kyoho” fruit were provided. Heterologous overexpression in Arabidopsis, transient overexpression and Tobacco rattle virus (TRV)-induced gene silencing (VIGS) assays in the grape berry were used to try to verify the function of the isolated putative PYR gene. The *VlPYL1* gene played an important role in fruit development, as up-regulation of the *VlPYL1* gene could promote anthocyanin accumulation in grape berry skin. Additionally, ABA-related genes, such as *ABF2* and *BG3*, could directly respond to *VlPYL1* expression levels. These results indicate that the ABA receptor VlPYL1 acts as a positive regulator to promote ripening in grapevine.

## Materials and Methods

### Plant Materials

The grape cultivar “Kyoho” (*Vitis vinifera* × *Vitis labrusca*) and strawberry plants (*Fragaria* × *ananassa* cv. “Hongyu”) were grown in a glasshouse at Shanghai Jiao Tong University, Shanghai, China (31°11′N, 121°29′W). Thirty vines of uniform “Kyoho” grapevines were selected. The cultivation medium was a mixture of sand, loam and perlite (1:1:1). All vines were placed at a spacing of 1.5 m × 2.0 m in north–south oriented rows, and the shoots were trained vertically with three shoots per vine. Vines were maintained under natural ventilated and light conditions throughout the growing season except on rainy days. Nutrition and irrigation management was conducted as described previously (Wang B. et al., 2012).

Strawberry plants were planted in pots (diameter, 250 mm; and depth, 250 mm) containing a mixture of soil, sand, and organic fertilizer (5:2:1, v/v/v). The seedlings were grown in growth chamber under the following conditions: 25°C, and a 12-h photoperiod with a photosynthetic photon flux density of 300 μmol m^-2^s^-2^.

The *Arabidopsis thaliana* (ecotype Columbia-0) seeds were sown on 1/2 Murashige and Skoog (MS) medium for 1 week, and then transferred in plastic basins filled with peat and vermiculite (1:1 by volume). The Arabidopsis plants were cultured in the controlled environment cabinets under LD (16 h light/8 h dark) conditions with a fluence rate of 200 μmol m^-2^s^-2^ of white light (produced by cool-white fluorescent lamps) at 22°C.

### Spatiotemporal and Tissue-Specific Expression of *VlPYL1*

Different developmental stages of grape fruit were collected at 7, 21, 28, 42, 56, 74, and 96 days after anthesis (DAA). Analysis was performed on three separate pools of c. 20 fruits each. Each pool was from three different plants. After removal of seeds, the pulp and peel were cut into small cubes of 0.5–0.8 cm^3^. Mixed samples of grape berries were harvested at each stage, while leaves, young apical shoot internodes (stems), flowers, and roots used for organ-specific expression analysis were collected at the flowering stage with three replicates. All samples were quickly frozen in liquid nitrogen and stored at -80°C until use. Quantitative reverse transcription PCR (RT-qPCR) primers to detect *VlPYL1* were designed using Primer Express 3.0.1. Primer sequences are shown in **Supplementary Table S1**.

### RNA Isolation and cDNA Synthesis

Total RNA was extracted from different developmental stages of grape, as well as root, stem leaf, and flower tissues using a TaKaRa MiniBEST Plant RNA Extraction Kit (Takara, Dalian, China). Genomic DNA was eliminated using a TaKaRa MiniBEST Plant Genomic DNA Extraction Kit (TaKaRa) followed by an RNA Clean Purification Kit (TaKaRa). The purity and integrity of RNA were analyzed by both agarose gel electrophoresis and NANODROP2000 (Thermo Scientific, Wilmington, DE, United States). To generate first-strand cDNA, 1 μg of total RNA was reverse-transcribed using the PrimeScriptTM RT reagent kit (TaKaRa) according to the manufacturer’s protocol.

### Gene Isolation and Sequence Analysis

The cDNA obtained above was used as a template for amplifying the full encoding length of *VlPYL1*. PCR was performed under the following conditions: 94°C for 90 s, 35 cycles at 94°C for 20 s, 57°C for 20 s, and 72°C for 30 s using the Fast Pfu Master DNA Polymerase (Novoprotein Scientific Inc.). PCR products were ligated into a pEASY-Blunt Simple vector and subsequently transformed into *Escherichia coli* DH5α. Positive colonies were selected, amplified, and sequenced by Tsingke China (Shanghai, China).

For promoter isolation, 1.5 kb upstream sequence of the *VlPYL1* start codon ‘ATG’ was cloned as promoter based on the grape gene library^1^ (Vitulo et al., 2014). Then the *VlPYL1* promoter was analyzed for *cis*-acting elements using the Plant-CARE database^2^. To identify the conserved regions, multiple sequence alignments were performed using ClustalX (version 1.83) (Thompson et al., 1997) and DNAMAN 6.0 (Lynnon Biosoft, Quebec, Canada). For phylogenetic analysis, MEGA 6.0 (Tamura et al., 2013) was used to construct maximum parsimony (MP) trees with the following parameters: Poisson model, pairwise deletion and bootstrap (1000 replicates; random seed).

### Quantitative Real-Time PCR Analysis

Primers used for quantitative real-time PCR (qPCR) were designed using Primer express 3.0.1 and are listed in **Supplementary Table S1**. The *VvActin* and *AtACT2* genes were used as internal controls for grape and *A. thaliana*, respectively. qPCR was performed on a CFX connect Real Time PCR Detection System (Bio-Rad) using the following program: 95°C for 30 s, followed by 40 cycles of 95°C for 5 s and 60°C for 10 s. The relative expression levels of the amplified products were analyzed using the comparative C_T_ method based on C_T_ values (Livak and Schmittgen, 2001).

### Subcellular Localization of *VlPYL1*

For subcellular localization of *VlPYL1*, the coding region of *VlPYL1* lacking its stop codon was cloned into the pHB vector containing the GFP reporter gene to produce a fusion construct under the control of the CaMV35S promoter (Li et al., 2016). The fusion construct was transferred into *Agrobacterium tumefaciens* strain GV3101 by heat shock. *Agrobacterium* mediated transient transformation of tobacco (*Nicotiana benthamiana*) leaves was done according to Sparkes et al. (2006). The cells of transformed tobacco leaves were observed with a laser scanning confocal microscope (Leica TCS SP5-II, Germany).

### Construction of Expression Vectors and Agroinfiltration

To construct a *VlPYL1* over-expression (*VlPYL1*-OE) vector, full-length cDNAs of the *VlPYL1* gene were amplified. The resultant PCR product was inserted into the pHB vector under the control of the double 35S-CaMV promoter.

For construction of silencing vectors, the tobacco rattle virus (TRV)-based pTRV1 and pTRV2 vectors used for RNAi (Dong et al., 2007) were kindly donated by Dr. Liu Yu-le, Qinghua University. A 420-bp cDNA fragment of *VlPYL1* (from 137 to 556 bp) and a 390 bp fragment of *VvPDS* (from 166 to 556 bp) were cloned into pTRV2, respectively (Dong et al., 2007).

*Agrobacterium tumefaciens* strain GV3101 containing pHB-*VlPYL1*, pTRV1, pTRV2, or the pTRV2 derivatives pTRV2-*VlPYL1* and pTRV2-*VvPDS* was grown at 28°C in Luria–Bertani medium supplemented with kanamycin and rifampin. When the culture reached an OD600 of approximately 1.0, *Agrobacterium* cells were harvested and resuspended in infection buffer (10 mM MgCl_2_, 10 mM MES, pH 5.8, and 100 μM acetosyringone) and shaken for 4 h at 28°C before being used for infiltration. *Agrobacterium*-mediated infiltration by syringe injection into strawberry and tobacco was performed as described by Dong et al. (2007), Chai et al. (2011), and Li Q. et al. (2013). Large green grape berries (45 DAA) were used and infected with a syringe until the whole fruit became hygrophanous. Approximately 2, 4, 6, and 8 days after treatment, the fruits infiltrated with the overexpression or the silencing constructs were harvested and used to detect the vector by reverse transcription PCR (RT-PCR). For analysis of phenotypes, 200 fruits were injected, with 50 fruits being transformed with *VlPYL1*-OE or *VlPYL1*-RNAi and the other 50 used as the control (i.e., injected with the pHB or TRV empty vector). For the expression analysis of ripening-related genes, three fruits were combined as an individual sample. The pulp and peels without seeds were frozen in liquid nitrogen and stored at -80°C until use.

### Color Measurement

The anthocyanin content in whole grape skins was measured after inoculation for 8 days and different growth periods at 20, 30, 40, 50, 60, 70, 80, and 90 DAA; Grape skin (50 mg) was pulverized with liquid nitrogen and then each sample was homogenized in 1% (v/v) hydrochloric acid in methanol and shaken at 4°C overnight. Anthocyanin concentrations were determined by measuring the absorbance of the extract at 530 nm using an ultraviolet–visible (UV-Vis) spectrophotometer (Pirie and Mullins, 1976; Lurie et al., 2009).

The color of grape berry skin was measured after inoculation for 8 days with a tristimulus reflectance colorimeter (S-6017, Chentaike, Beijing, China). Color parameters were recorded as *L*^∗^ (lightness), *a*^∗^ (redness) and *b*^∗^ (yellowness).

### ABA and Drought Tolerance Assay in Transgenic Arabidopsis

The sense vector was genetically introduced into Arabidopsis using *Agrobacterium*-mediated transformation as described in a previous report (Zhang et al., 2006). Transformants were selected based on their resistance to hygromycin (Hyg). Positive transgenic seedlings were grown in pots containing a mixture of soil and vermiculite (1:2, v/v) to select for T2 and T3 seeds. After surface sterilization of the seeds, approximately 100 seeds of each genotype were sowed on 1/2 MS medium supplemented with 1.5% sucrose and 0.7% agar. Seeds were vernalized at 4°C for 3 days and then incubated in a growth chamber at 22°C with a 16 h light and 8 h dark photoperiod.

Surface-sterilized seeds were sown on 1/2 MS without or with ABA (1 and 3 μM) or mannitol (100 mM). Seeds were scored as germinated after 48 h and 4 days. The germination rate was determined as the radicle emerges from the seed coat.

For ABA and mannitol treatment, 5-day-old seedlings were plated on 1/2 MS medium and 1/2 MS medium supplemented with ABA (1 and 3 μM) or mannitol (200 mM) for 8 days, and then their root lengths and leaf areas were measured using PC-aided image processing software (Bakr, 2005). The treated plants were harvested at the indicated time points, immediately frozen in liquid nitrogen, and stored at -80°C until used for RNA extractions.

### Statistical Analysis

Data were analyzed using variance (ANOVA), and the averages were compared by the Duncan’s New Multiple Range test or *T*-test (*P* < 0.05) using SPSS 17.0 (SPSS, United States).

## Results

### Phylogenetic and Identification Analysis of *VlPYL1*

Three genes, named *VvPYL1*, *VvPYL2* and *VvPYL3* were cloned from *Vitis vinifera* cv. Muscat of Hamburg (Li et al., 2012). To clone the *VlPYL1* gene in “Kyoho” (*Vitis vinifera* × *Vitis labrusca*), the nucleotide and protein sequences of PYR1 (At4G17870) and PYL1 (At5g46790) in Arabidopsis, PYR1 in strawberry (No. JF268669) and *VvPYL1* (LOC100267793) (Li et al., 2012) were used to BLAST to a grape gene library^3^ (Vitulo et al., 2014), and 8 significantly highly similar proteins with conserved amino acid regions were obtained. In agreement with Boneh et al. (2012b), the phylogenetic analysis results showed that these proteins from the three species could be classified into three large groups: in one group was the *RCAR7* protein, which was most closely related to *AtPYR1*, *AtPYL1* and *FaPYR1*, followed by gene VIT 204s0008g00890.1, which is annotated as “abscisic acid receptor *PYL2/RCAR8*” (**Supplementary Figure S1B**). Although the *RCAR7* showed higher homology to *AtPYR1* and *FaPYR1* than *AtPYL1*, the name *VlPYL1* was used in consistency with naming by Li et al. (2012).

Based on the nucleotide sequence, gene-specific primers (**Supplementary Table S1**) were designed to amplify a coding sequence of the *VlPYL1* gene from “Kyoho” grape fruit. A 645 bp cDNA was isolated from grape fruit through RT-PCR (**Supplementary Figure S2**). The cDNA included an open reading frame encoding a deduced protein of 215 amino acids (**Supplementary Figure S3**), in which the putative conserved domains were detected by homology analysis (protein BLAST on the NCBI website^4^) (**Supplementary Figure S1A**), which suggested that the putative grape ABA receptor gene, *VlPYL1*, was isolated successfully. *VlPYL1* protein exhibited 74.4% sequence identity with the Arabidopsis *AtPYR1* protein. *VlPYL1* and strawberry *PYR1 (FaPYR1)* shared 72.35% sequence similarity (**Supplementary Figure S3**).

*VlPYL1* promoter sequence was cloned and sequenced by sanger method (GenBank Accession Number MG917083). To define *cis*-acting elements in the *VlPYL1* promoter, a motif search was conducted using the Plant-CARE databases. As illustrated in **Supplementary Table S2**, two ABRE (ABA-responsive element) motifs with the core sequences GCAACGTGTC and TACGTG could be identified. The sequence analysis suggested that several *cis*-elements, including defense and stress responsiveness (TC-rich repeats), fungal elicitor responsive element (W-box, which is recognized by the WRKY protein), cold response (LTR), and element essential for the anaerobic induction (TGGTTT) are also present in the *VlPYL1* promoter sequence (**Supplementary Table S2**). The sequence also contains hormone responsive *cis*-acting elements such as methyl jasmonate (MeJA) responsive TGACG -motif, ethylene responsive element, and salicylic acid responsive elements.

### Subcellular Localization and Expression Pattern of *VlPYL1*

To provide more insights into the function of *VlPYL1*, the subcellular localization of the protein was investigated using a full-length *VlPYL1* N-terminal GFP fusion protein. The fusion gene of *VlPYL1-GFP* was transformed into tobacco (*Nicotiana benthamiana*) leaves using *A. tumefaciens* infiltration. As shown in **Supplementary Figure S4**, the *VlPYL1*-GFP fusion protein localized in both the nucleus and cytosol.

**FIGURE 1:**
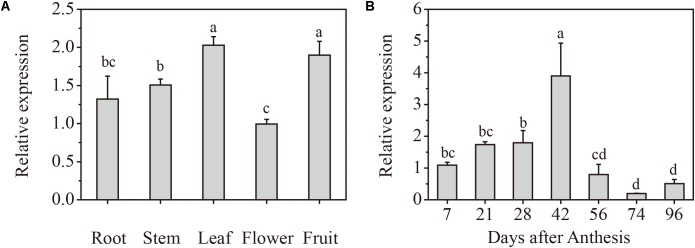
Spatiotemporal pattern of *VlPYL1* expression in grape fruits. **(A)** RT-qPCR analysis of *VlPYL1* expression in different organs of the “Kyoho” grape plant. **(B)** Changes in the mRNA expression level of the *VlPYL1* gene in “Kyoho” grape fruits. *VlPYL1* transcripts were detected by RT-qPCR in the pulp and peel during grape fruit development. Actin mRNA was used as an internal control. The error bars represent the SD (*n* = 3). Different letters indicated a statistical difference at *P* < 0.05 as determined by Duncan’s multiple range test.

To determine the expression profile of *VlPYL1* in grapes, total RNA was extracted from roots, stems, leaves, flowers, and fruits and reverse-transcribed into cDNA. Based on the *VlPYL1* gene sequence, a 152 bp *VlPYL1* gene specific fragment was used to carry out the RT-qPCR to identify expression patterns. The results showed that *VlPYL1* was abundant in grape leaves and fruits, followed by stems and it showed a low level in roots and flowers (**Figure 1A**).

To investigate whether *VlPYL1* is involved in grape fruit development, the levels of its transcript during berry ripening were observed. The results showed that *VlPYL1* was expressed in both green and purple fruits. The transcripts of the *VlPYL1* gene increased gradually coupled with green fruit growth and enlargement (from 7 to 42 DAA). However, transcripts decreased rapidly at the veraison stage (56 DAA), and reached their lowest level in ripe fruits (74 and 96 DAA) (**Figure 1B**).

### Overexpression of *VlPYL1* in Arabidopsis Leads to the ABA and Osmotic Stress Hypersensitive Phenotype During Germination

The *VlPYL1* overexpression (*VlPYL1*-OE) vector was introduced into Arabidopsis plants, and four transgenic lines were obtained. The T3 homozygous 1-1 and 2-4 lines with high and moderate levels of expression of VlPYL1, respectively, were chosen for functional analysis (**Supplementary Figure S5**).

**FIGURE 2:**
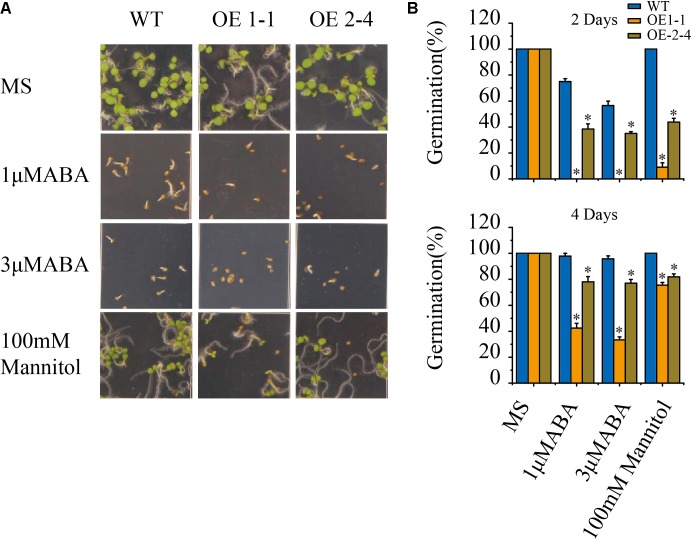
Seed germination phenotypes of 2 × 35S:: *VlPYL1* transgenic *Arabidopsis* plants. **(A)** ABA and osmotic stress hypersensitive inhibition of germination in 2 × 35S:: *VlPYL1* transgenic *Arabidopsis* lines compared with wild-type plants. Seeds were grown on MS medium with or without 1 μmol ABA, or 3 μmol ABA, or 100 mM mannitol. Photographs were taken 4 days after sowing. **(B)** Percentage of seed germination in the presence of the stress treatment. Germination percentage is determined at 2 (upper graph) and 4 days (lower graph) after seeds are scored on MS, 1 μmol ABA, 3 μmol ABA, or 100 mM mannitol. Data are averages ± SD from three independent experiments (n∼50 seeds per experiment). ^∗^*p*-value < 0.05 when comparing data for each genotype versus the wild-type under the same conditions.

We treated the 1-1 and 2-4 lines and wild type with 1 and 3 μM ABA and then investigated their seed germination phenotypes. No significant differences in seed germination rate between *VlPYL1*-OE plants and wild type were found under control conditions. However, the seed germination rates of the two *VlPYL1*-OE transgenic Arabidopsis lines were less than the wild type in the presence of 1 μM ABA and 3 μM ABA (**Figures 2A,B**).

Since the ABA sensitivity at the seed germination stage of *VlPYL1*-OE transgenic Arabidopsis plants have been enhanced, we speculated that overexpression of *VlPYL1* may also affect plant osmotic stress tolerance. To test this, the seed germination of *VlPYL1*-OE transgenic Arabidopsis lines under 100 mM mannitol treatment was analyzed. Similar to the ABA treatment, the germination greening ratio and germination rate of *VlPYL1* transgenic lines were significantly inhibited by 100 mM mannitol, but the inhibition in the wild type was less severe (**Figures 2A,B**).

These results demonstrate that *VlPYL1*-OE seeds exhibit a sensitivity to inhibition of germination by exogenous ABA and osmotic stress.

### Overexpression of *VlPYL1* in Arabidopsis Confers ABA Hypersensitive Phenotype and Promotes Plant Root Growth to Osmotic Stress During Seedling Growth

**FIGURE 3:**
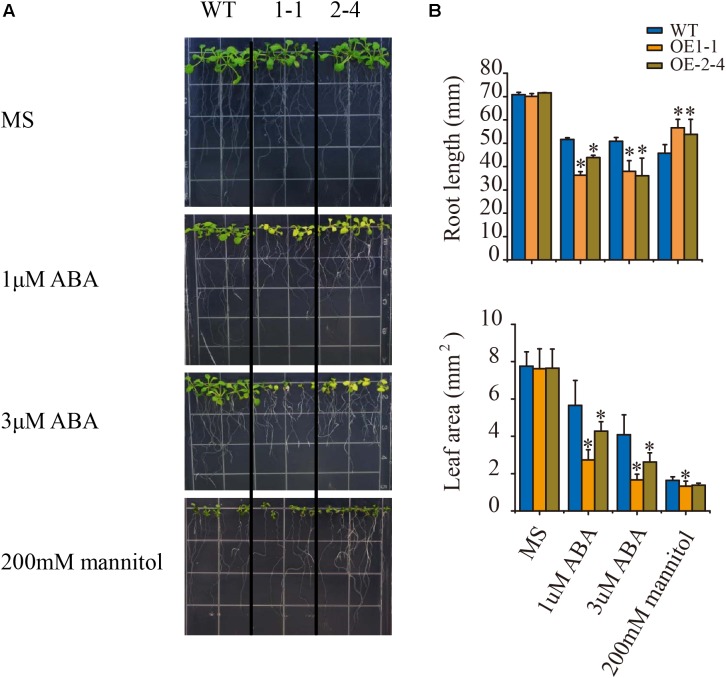
ABA hypersensitive inhibition of root growth in 2 × 35S:: *VlPYL1* transgenic *Arabidopsis*. **(A)** Growth of Col-0, 2 × 35S:: *VlPYL1*, two independent 2 × 35S:: *VlPYL1* lines (1-1, 2-4) in medium (MS) supplemented with 1 and 3 μM ABA. Photographs of representative seedlings 8 days after the transfer of 5-day-old seedlings from MS to plates supplemented with ABA. **(B)** Root length and leaf area of WT and two transgenic lines on 1 μM ABA, 3 μM ABA, or 100 mM mannitol.

ABA acts as a repressor in root growth, and root growth under ABA treatment is important indicator to evaluate plant ABA sensitivity (Zhang et al., 2013a). To fully examine the plant phenotype under ABA treatment, we sowed wild type and the two transgenic lines on MS medium. After 5 days, plants were transferred to vertical plates and supplemented with 0, 1, or 3 μM ABA for 8 days (**Figure 3A**). When grown on plates without ABA, the plants displayed no visible root development phenotype. However, the two transgenic lines showed significantly reduced root length and leaf area comparing with the wild-type plants on the plates with the supplement 1 or 3 μM ABA (**Figures 3A,B**). These results indicated that *VlPYL1* overexpression lines were hypersensitive to ABA-mediated inhibition of root growth in Arabidopsis.

To extend the osmotic stress-tolerance analysis, 5-day-old seedlings were transferred to 1/2 MS plates without (control) or with 200 mM mannitol for 8 days. In the presence of 200 mM mannitol, the shoot growth of *VlPYL1*-OE plants was inhibited slightly compared to that of the control, but transgenic Arabidopsis plants displayed longer primary roots than wild type (**Figures 3A,B**).

### Analysis of ABA-Responsive Gene Expression in Transgenic Arabidopsis Plants

**FIGURE 4:**
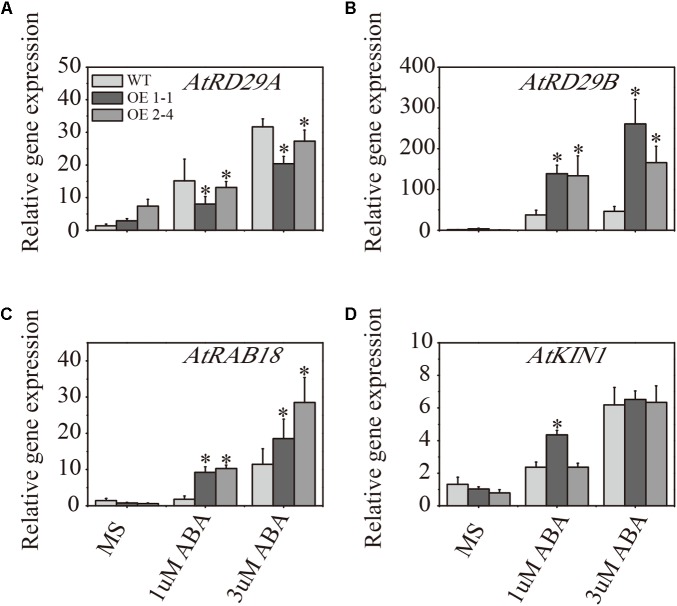
Expression analysis of ABA-regulated genes in 2 × 35S::*VlPYL1* transgenic *Arabidopsis* compared to wild-type. The mRNA levels of the indicated genes, including *RD29A* **(A)**, *RD29B* **(B)**, *RAB18* **(C)**, and *KIN1* **(D)** are determined by RT-qPCR analysis using total RNAs isolated from mock-treated *Arabidopsis* or ABA-treated plants. Data are averages ± SD from three independent experiments. Expression of β-*ACTIN2* was used to normalize data. ^∗^*p*-value < 0.05 indicate statistically significant expression change of transgenic lines w.r.t. respective WT in different treatments.

To examine whether the enhancement of ABA sensitivity in transgenic plants was accompanied by altered mRNA levels of the stress-responsive genes, the abiotic stress-responsive genes *RD29A*, *RD29B*, *RAB18* and *KIN1* mRNA levels were determined by RT-qPCR analysis using total RNAs isolated from mock-treated Arabidopsis or ABA-treated plants for 8 days (Zhang et al., 2013a). As shown in **Figure 4**, the transcript levels of most of these stress marker genes were higher in ABA stress plants compared with plants grown in the MS medium without ABA treatment. Compared with the WT lines, both *AtRD29B* and *RAB18* exhibited a significantly higher level of expression in transgenic lines following ABA treatment. Notably, the expression level of *AtRD29A* was slightly higher in WT plants than in transgenic plants under ABA stress. Expression of *KIN1* was significantly higher in 1-1 lines under 1 μM ABA treatment, but both transgenic plants expressed similar levels under 3 μM ABA treatment (**Figure 4**).

### Over-Expression of *VlPYL1* Alters the Developmental Processes of Grape Fruits

Developmental and ripening parameters were characterized (**Supplementary Figure S6**). The earliest sample stage corresponded with the green-hard stage characterized by berries at least 11 mm in diameter with a minimum 6° Brix. An increasing trend in weight, size, soluble solids, and anthocyanin were consistent with the phase transition from green-hard (0 DAA) to green-soft (50 DAA), pink-soft (60 DAA), and red-soft (80 DAA) developmental stages (**Supplementary Figure S6**).

**FIGURE 5:**
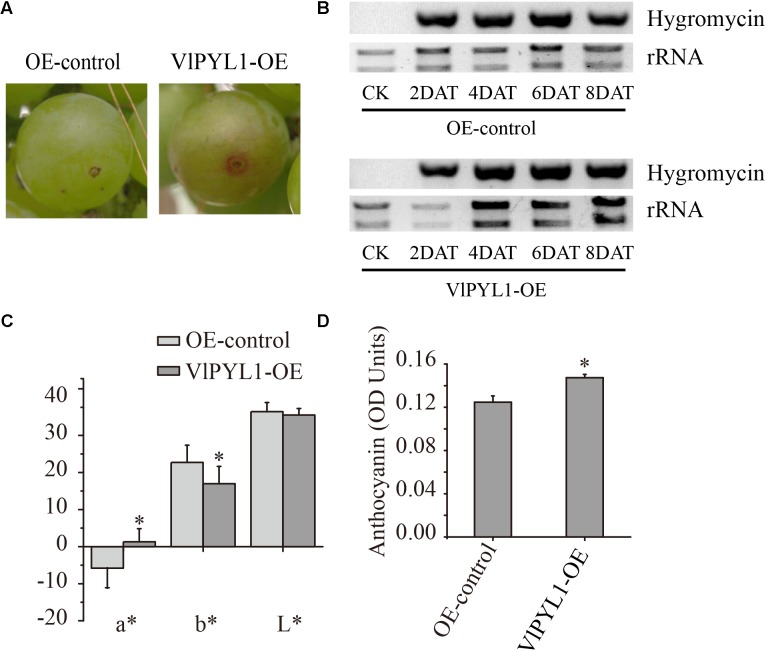
**(A)** “Kyoho” fruits agroinfiltrated with empty vector (EV) and agroinfiltrated fruit with the *VlPYL1-OE* construct (overexpression) at 6 days after injection. **(B)** RT-PCR analysis of OE vector expression in fruits. Hygromycin gene (resistance marker gene) expression was detected in fruits infiltrated with Agrobacterium containing the OE vector (harvested on day 2, 4, 6, and 8 post-injection) but was not detected in fruits infiltrated with Agrobacterium alone (lane 1). **(C)** Color parameters (L^∗^, a^∗^, b^∗^) of the control and overexpression fruits. **(D)** The total anthocyanin content was measured in the control and overexpression fruits. Asterisks indicated statistically significant differences at *P* < 0.05 as determined by Student’s *t*-test.

Big green grape berries (45 days after anthesis) were selected for injection with the empty vector pHB and *VlPYL1*-OE with a 1-ml syringe. After being infiltrated for 8 days, the *VlPYL1*-OE-infiltrated berries showed faster color development compared with the empty vector pHB infiltrated berries (**Figure 5A**). A significant increase in the red color (*a*^∗^), a decrease in the yellowness (*b*^∗^) and similar lightness (*L*^∗^) values were obtained for OE fruits as compared to the control fruits. Fruit anthocyanin contents also increased in OE fruits compared with the control fruits (**Figures 5C,D**). The number of *VlPYL1*-OE berries with the observed red spot was higher than control berries 8 days after injection (**Supplementary Table S3**). However, the accumulation began to converge in the *VlPYL1-OE* and control berries 16 days after infiltration (**Supplementary Tables S3**, **S4**).

To confirm the results caused by *VlPYL1*-OE in grape berries, a pHB-specific primer for a 752 bp amplicon of the vector were designed. As shown in **Figure 5B**, the PCR products could be detected accompanying the color development in the infiltrated fruits. These results confirmed that over-expression of *VlPYL1* could alter the developmental processes of grape fruits.

### Overexpression of the *VlPYL1* Gene Alters a Set of ABA-Responsive and Flavonoid Biosynthesis-Related Gene Transcripts

**FIGURE 6:**
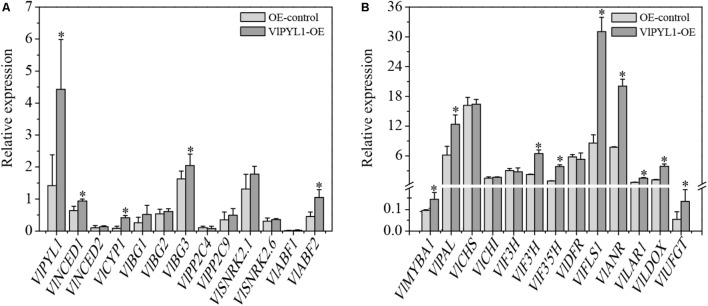
Transcriptional changes of the ABA signaling genes and flavonoid biosynthesis-related genes in *VlPYL1-OE* fruits. **(A)** The mRNA expression levels of ABA signaling genes in *VlPYL1* fruit and control fruit were detected by RT-qPCR. **(B)** Relative expression by RT-qPCR of flavonoid biosynthetic pathway genes in the grape fruits of *VlPYL1* overexpression fruits or control solution. Data are averages ± SD from three independent experiments. Expression of *VlActin* was used to normalize data. Asterisks indicate significant differences by *t*-test analysis: ^∗^*P* < 0.05.

Here, the mRNA expression levels of the ABA-responsive genes, including ABA synthesis-associated genes *NCED1*, *NCED2*, *BG1*, *BG2* and *BG3*, ABA degradation-related gene *CYP1*, ABA signal transduction associated genes *PP2C9*, *SnRK2*.1, *SnRK2*.6, *ABF1* and *ABF2* were analyzed by RT-qPCR (**Figure 6A**). The results showed that *PYL1*, *NCED1*, *CYP1*, *BG3* and *ABF2* were significantly up-regulated in the *VlPYL1*-OE grape fruits.

To explore the relationship of anthocyanin biosynthesis and *VlPYL1*, the mRNA levels of the flavonoid upstream pathway gene (*PAL*), the flavonoid biosynthesis-related genes (*CHS*, *CHI*, *F3H*, *F3′H*, *F3′5′H*, *DFR*, *LDOX*, *UFGT*, *FLS1*, *LAR1*, and *ANR*) and a regulatory gene (*MybA1*) were also analyzed by RT-qPCR (**Figure 6B**). As shown in **Figure 6B**, the mRNA expression levels of *MYBA1*, *PAL*, *F3′H*, *F3′5′H*, *FLS1*, *ANR*, *LAR1*, *LDOX* and *UFGT* were significantly up-regulated in the *VlPYL1*-OE grape berries.

### Transient Expression of *VlPYL1*- RNAi Vector Mediated by Agrobacterium

After being infiltrated for 12 days, the pTRV2-*VlPYL1* infiltrated grape berries and controls were both beginning to turn into the veraison stage (**Supplementary Figure S7A**). However, the strawberry fruits infiltrated with pTRV1 and pTRV2-*VlPYL1* showed an arrest of color development (Chai et al., 2011) compared to the control strawberry fruits (**Supplementary Figure S8A**). Further analysis of *FaPYR1* transcripts by RT-qPCR was performed in the receptacle of independent fruits injected with either the silencing construct or the empty vector. As shown in **Supplementary Figure S8B**, *VlPYL1*-RNAi resulted in a dramatic decrease in *FaPYR1* transcript level. To test whether the TRV-vector can directly infect grape berries, a TRV-RNA1-specific primer for a 647 bp amplicon and a TRV-RNA2-specific primer for a 367 bp amplicon were designed. RT-PCR analysis of the vector transcripts were performed in the control and RNAi grape berries. As shown in **Supplementary Figure S7B**, PCR products were only detected in 2-day-old and 4-day-old TRV-infiltrated berries, but could not be detected 6 days after infiltration. In addition, we first cloned the CDS sequence of *PDS* (PHYTOENE DESATURASE) in grape and then silenced it. Photo-bleaching was shown on the newly developed leaves 2 weeks after agroinoculation in tobacco, but not in grape leaves, also indicating that TRV vector cannot be used to silence genes in “Kyoho” grapes (**Supplementary Figures S7C,D**).

## Discussion

*PYL* family genes encode receptors that are involved in ABA signal transduction (Ma et al., 2009; Park et al., 2009). Most studies on ABA receptors have focused on Arabidopsis, whereas only a few of these receptors have been characterized in other plants. Here, we report an *in planta* functional characterization of a grape *PYL* which is functional and translates the ABA signaling during osmotic stress or ABA treatment in Arabidopsis. Consistent with findings from Boneh et al. (2012b), phylogenetic and sequence analysis revealed that *RCAR7/VlPYL1* amino acid sequence was highly similar to the members of the well-established ABA-related Arabidopsis PYR/PYL/RCAR family, belonging to group III of grape *PYL* (**Supplementary Figures S1B**, **S3**). Two different bases did not lead to the change in amino acid between VlPYL1 from “Kyoho” grape and VvPYL1 from *Vitis vinifera* cv. Muscat of Hamburg (**Supplementary Figure S2**). It seemed that the expression level of *VvPYL1* of Muscat of Hamburg in leaf and stem was higher than in root (Li et al., 2012). Similarly, “Kyoho” *VlPYL1* showed high expression in the leaf and fruit, which seems to support a relevant role for ABA perception. Moreover, subcellular localization suggested that the *VlPYL1* protein resided in both the nucleus and the cytosol in agreement with previous result (Li et al., 2012). This outcome is similar to the pattern seen in Arabidopsis (Park et al., 2009) and gives the evidence to support the cellular function of *VlPYL1* involves signaling components in both the nucleus and cytosol. Moreover, *in silico* promoter analysis revealed the presence of two ABA responsive elements (ABRE) that were also found within 1.5 kb upstream sequence of *VlPYL1* (**Supplementary Table S2**). These observations indicated a functional role of this grape *PYL1* in the ABA and abiotic stress signaling network. Two independent homozygous Arabidopsis transgenic lines constitutively expressing *VlPYL1* were analyzed for ABA sensitivity and stress tolerance on MS-based media. It showed that *VlPYL1* overexpression in Arabidopsis results in ABA and osmotic stress hypersensitive seed germination. Moreover, when grown on plates supplemented with 1 and 3 μM ABA, transgenic lines showed statistically significant differences compared with WT, and both transgenic lines showed significantly reduced root length and leaf area (**Figure 4B**). The genetic evidence suggested that *VlPYL1*-OE plants were sensitive to ABA similar to the *PYL*s in Arabidopsis (Ma et al., 2009; Santiago et al., 2009; Lee et al., 2013) and that it also acts as positive regulator of ABA signaling. Moreover, in this study, the stress marker genes such as *RD29B* and *RAB18* were significantly up-regulated in *VlPYL1*-OE lines in comparison to WT under exogenous ABA treatment. It has been reported that these stress marker genes were regulated through ABA signaling during the abiotic stress response (Lang and Palva, 1992; Jakab et al., 2005; Li F. et al., 2013). And AtRD29B was function in the ABA-dependent pathway, whereas AtRD29A was function in the ABA-independent pathway (Shinozaki and Yamaguchi-Shinozaki, 2000). Also, the *RD29B* promoter was highly responsive to salt stress, whereas the *RD29A* promoter was more sensitive to drought and cold stresses (Msanne et al., 2011). The variable expression patterns of stress markers such as *RD29A* and *RD29B* (**Figure 4C**) could be attributed to different ABA responsive element in promoter and multiple layers of gene expression regulation. Key transcription factors such as DREB and AREB, which ultimately control the expression of stress genes such as RD29A and RD29B, are controlled by VlPYL1 or other components such as PP2C (since PP2C is downstream of the PYLs) or SnRK2s, which are known to regulate the stress-responsive transcription factors in dose-dependent way (Gonzalez-Guzman et al., 2012). It may be speculated that VlPYL1 might interact with and regulate some critical components of the ABA signal transduction pathway such as Ser/Thr kinases, especially PP2C (Park et al., 2009; Li W. et al., 2013; Pizzio et al., 2013); these kinases have been implicated in ABA stress and could influence the expression of stress-responsive genes to regulate the physiological process.

The grape berry ripening process is non-climacteric and does not rely on a sole ethylene signal. Numerous studies have highlighted ABA as an important hormone in the inception and color development stages of fruit ripening (Peppi et al., 2006; Cantin et al., 2007; Ferrara et al., 2015). However, the molecular mechanism of ABA in the regulation of grape fruit development, especially fruit maturity, is still under question. The PYR/PYL/RCARs have been suggested to play a role in grape fruit ripening (Jia et al., 2017), but substantial evidence has been lacking. Here, we also provided evidence to determine the role of PYLs on anthocyanin accumulation in grape. A previous study reported that downregulation of *FaPYR1* transcripts by RNA interference (RNAi) inhibited fruit abnormal reddening. This loss of red coloring in *FaPYR1* RNAi fruits could not be rescued by exogenously applied ABA (Chai et al., 2011), indicating that *PYL* was a necessary regulator of fruit ripening. Similar result with the previous reports of *FaPYR1* (Chai et al., 2011), *CsPYL1* and *CsPYL2* (Wang Y. et al., 2012) and all of the *MaPYL*s (except for *MaPYL1*) (Zhu et al., 2017), the expression of *VlPYL1* kept higher level during the early stages of fruit development suggested that it might participate in the regulation of fruit development. However, the expression changes of *PYR1* in grape cv. Fujiminori were consistent with ABA content which increased rapidly in the former phase and stay high level at 14 weeks post flowering (Jia et al., 2017). Genetic characteristics of the plants may lead to the different expression patterns of *PYL1* between “Fujiminori” and “Kyoho” grapes. Other PYR/PYL/RCAR family members in Kyoho may contribute to the ABA signal transduction after veraison stage. Obviously, *VlPYL1* transcripts decreased rapidly just before the veraison (**Figure 1B**), whereas anthocyanin content began rapidly increasing after the veraison stage (**Supplementary Figure S6D**). It suggested that the *VlPYL1* might be mainly involved in “Kyoho” early fruit development and stimulation of ripening initiation.

Stable transformation is a method of assessing the functional significance of transgenes, but the stable transformation system is time-consuming and requires great care in handling of the experiment. On the other hand, the transient gene expression system allows for rapid *in vivo* analysis of genes in plants such as strawberry (Guidarelli and Baraldi, 2015). Many studies have successfully applied transient gene expression system using agroinfiltration to grape leaves (Jelly et al., 2014), but no similar technique has been reported in grape berries. In this study, we applied a transient gene expression assay to analyze the effects of gene expression due to overexpression of ABA receptor gene in grape fruits. Over-expression of the *VlPYL1* gene in grapes led to more *PYL1* transcript (**Figure 6A**), and the *VlPYL1*-OE fruit exhibited an increase of anthocyanin accumulation compared to the control fruit. **Figure 3C** also shows that over-expression of the *VlPYL1* gene improved color of the grape skin due to higher values of a^∗^. Furthermore, the transcripts of the up-regulated ABA-dependent genes, including *NCED*, *BG3* and *ABF2*, and pigment-related genes, including *PAL*, *UFGT* and *MYBA1*, were up-regulated in the *VlPYL1*-OE berries, in which the mRNA expression level of *VlPYL1* was up-regulated threefold (**Figure 6A**). A previous study demonstrated that *VvABF2* was involved in the regulation of grape fruit ripening (Nicolas et al., 2014), and high expression of *VvBG1* suggested a regulatory role in grape berry ripening (Sun et al., 2015). It is also interesting to note that *FaBG3* was a positive regulator of strawberry fruit ripening (Li Q. et al., 2013). These results were consistent with a positive role of *VlPYL1* in ABA signaling during fruit ripening.

The transcript levels of *VlPAL*, *VlF3’H*, *VlF3’5’H*, *VlFLS1*, *VlANR*, *VlLAR1*, *VlLDOX*, *VlUFGT* and *VlMYBA1* were significantly up-regulated in the *VlPYL1*-OE berries, suggesting that *VlPYL1* may affect anthocyanin biosynthesis through regulating the expression of these flavonoid biosynthetic pathway genes. In the phenylpropanoid biosynthesis pathway, PAL is the first and rate-limited enzyme that contributes to the biosynthesis of anthocyanins, stilbenes and tannins. In addition, similar to other plants (such as strawberry), in both the field grown grapes and the *in vitro* experiments, *VvPAL* could be induced by exogenous ABA and directly influenced the accumulation of flavonols and anthocyanins (Hiratsuka et al., 2001a,b; Gagne et al., 2006). It is likely that the synthesis of more flavonols was also beneficial to *VlPYL1*-OE berries resulting from ∼3-fold higher expression levels of the *FLS1* gene (Downey et al., 2003). Higher expression levels of *LAR1* and *ANR* also indicated that these genes were favored in the enhanced synthesis of proanthocyanidins in *VlPYL1*-OE berries (Xie and Dixon, 2005). *UFGT* encodes a crucial enzyme that mediates carbon flow to anthocyanins in grapes (Boss et al., 1996). UFGT has been implicated in grape berry coloration (Ageorges et al., 2006; Castellarin et al., 2007), and overexpression of the *VVMYBA1-2* gene could increase the transcript level of *UFGT* (Cutanda-Perez et al., 2009). In grapes, *MYB1* was reported to regulate the anthocyanin synthesis pathway (Kobayashi S. et al., 2005; Walker et al., 2007; Azuma et al., 2008), and ABA has been reported to induce *VVMYBA1* expression (Jeong et al., 2004; Azuma et al., 2015). Since F3′H regulates the synthesis of cyanidin-type anthocyanins and F3′5′H, diverting the flux to synthesis of delphinidin derivatives, expression profiles of these two genes in concert with increased expression of *LDOX* and *UFGT* and *MybA1* would ensure the flux of flavonoid intermediates toward the synthesis of five main anthocyanidin aglycones including delphinidin, cyanidin, peonidin, petunidin and malvidin in *VlPYL1*-OE berries (Wang B. et al., 2012). These results suggested that the ABA signaling pathway initiated and controlled by PYL1 could activate the expression of the anthocyanin biosynthetic genes and accelerate the process of grape skin coloring.

## Conclusion

In this study, the *VlPYL1* gene was isolated from “Kyoho” grape fruits, and its biological role was assessed in fruits attached to the plant. Overexpression of *VlPYL1* significantly promoted anthocyanin accumulation in grape berry skins. Transient overexpression affected expression of the ABA pathway genes, increasing the transcript level of genes closely related to anthocyanin biosynthesis. The results showed that *VlPYL1* exerts a regulatory effect at the transcriptional level in a regulatory effect of ABA pathway aimed at promoting anthocyanin production in “Kyoho” grape berry skins. These data provide experimental information that supports the biological role of *VlPYL1* on the regulation of anthocyanin accumulation in grape berry skin as previously reported for *FaPYR1* in strawberry. In addition, we have demonstrated that TRV VIGS vectors could not be used to suppress functional genes in “Kyoho” grapes.

## Author Contributions

SS, WX, CZ, SW, and CM contributed to the project design. ZG performed the experiments and conducted statistical analysis. QL, YC, ML, HL, YW, JW, SD, and LW took part in the experimental work. ZG and JL wrote the manuscript. SW and CM revised the manuscript. All the authors read and approved the final manuscript.

## Conflict of Interest Statement

The authors declare that the research was conducted in the absence of any commercial or financial relationships that could be construed as a potential conflict of interest.

## Footnotes

1 http://genomes.cribi.unipd.it/grape/

2 http://bioinformatics.psb.ugent.be/webtools/plantcare/html/

3 http://genomes.cribi.unipd.it/grape/

4 http://blast.ncbi.nlm.nih.gov/Blast.cgi

## Supplementary Material

FIGURE S1 Sequence analysis of *VlPYL1*. **(A)** Finding conserved domains in the VlPYL1 protein. Conserved domains were determined for the *VlPYL1* protein via a 215-amino acid polypeptide BLAST in the NCBI database (http://blast.ncbi.nlm.nih.gov/Blast.cgi). The PYR/PYL/RCAR-like family belongs to the SRPBCC (START/RHO-alpha-C/PITP/Bet-v1/CoxG/CalC) domain superfamily of proteins that binds hydrophobic ligands. SRPBCC domains have a deep hydrophobic ligand-binding pocket. **(B)** Phylogenetic tree showing the relationship between VlPYL1 and other PYR/PYL/RCAR proteins. The tree presented here is a Neighbor–Joining tree based on an amino acid sequence alignment. The numbers next to each node give bootstrap values for 1000 replicates. Genes and accession numbers are as follows: *AtPYR1*, At4g17870; *At PYL1*, At5g46790; *At PYL2*, At2g26040; *At PYL3*, At1g73000; *At PYL4*, At2g38310; *At PYL5*, At5g05440; *At PYL6*, At2g40330; *At PYL7*, At4g01026; *At PYL8*, At5g53160; *At PYL9*, At1g01360; *At PYL10*, At4g27920; *At PYL11*, At5g45860; *At PYL12*, At5g45870; *At PYL13*, At4g18620; *FaPYR1*, JF268669; *RCAR1*, VIT 215s0046g01050; *RCAR2*, VIT 216s0050g02620; *RCAR3*, VIT 202s0025g01340; *RCAR4*, VIT 210s0003g01335; *RCAR5*, VIT 208s0058g00470; *RCAR6*, VIT 213s0067g01940; *VlPYL1/RCAR7*, VIT 202s0012g01270 and *RCAR8*, VIT 204s0008g00890.

FIGURE S2 Coding-sequence analysis of *VlPYL1* from “Kyoho” grape and *VvPYL1* from *Vitis vinifera* cv. Muscat of Hamburg reported by Li et al. (2012).

FIGURE S3 Amino acid sequence analysis of VlPYL1. Sequence alignment of the VlPYL1 protein with PYR1 and PYL1 in *Arabidopsis* and PYR1 in strawberries. Positions with identical amino acid residues are highlighted in blue, while similar amino acid residues are colored in pink and cyan.

FIGURE S4 Subcelluar localization of *VlPYL1* in tobacco leaves. *Nicotiana benthamiana* leaves were transiently infiltrated with *A. tumefaciens* GV3101 containing vectors expressing 2 × 35S::eGFP and 2 × 35S:: *VlPYL1-eGFP*. Confocal images were captured 72 h after inoculation. GFP image fluorescences are shown in the left panels, bright field images are shown in the middle panels, and overlap images are shown in the right panels.

FIGURE S5 Expression analysis of the *VlPYL1* gene in transgenic *A. thaliana* plants and WT. The *A. thaliana* actin gene was used as an internal control. ^∗∗^*p*-value < 0.01 when comparing data for each genotype versus the wild-type.

FIGURE S6 Morphological and physiological changes in “Kyoho” fruits during development. **(A)** Changes in fruit size and color, **(B)** berry weight (means ± SD, *n* = 30), **(C)** total soluble solids (means ± SD, *n* = 30), **(D)** anthocyanin content. Vertical bars represent SD of the values. Different letters indicated a statistical difference at *P* < 0.05 as determined by Duncan’s multiple range test.

FIGURE S7 **(A)** Effect of TRV-*VlPYL1* on grape fruit ripening. Note that both the control fruit and the RNAi fruit became red. The total anthocyanin content was measured in the control and TRV-RNAi fruits. **(B)** RT-PCR analysis of TRV expression in fruits. After infiltration, virus vector gene expression of both 647 bp pTRV1 and 367 bp pTRV2 was detected in fruits infiltrated with Agrobacterium containing TRV (lane 2 and lane 3: TRV-*VlPYL1*-treated grape fruits 2 and 4 days after infiltration) but were not detected in fruits infiltrated with Agrobacterium alone (lane 1) nor in TRV-*VlPYL1*-treated grape fruits 6 days (lane 4) and 8 days (lane 5) after infiltration. **(C)** TRV-VvPDS-treated grape plants 30 days after infiltration. **(D)** Mock-treated and TRV-*VvPDS*-treated tobacco plants 16 days after infiltration. The photobleached phenotype was observed in the plant (left) infiltrated with TRV-*VvPDS*, while the plant treated with TRV alone (right) remained green.

FIGURE S8 **(A)** Strawberry fruits agroinfiltrated with empty vector (EV) and agroinfiltrated fruit with *VlPYL1*-RNAi construct (RNAi) at 7 days after injection. **(B)** *FaPYR1* expression by RT-qPCR of strawberry receptacle infiltrated with empty vector (Control) and *VlPYL1*-RNAi vector, 7 days after injection. Error bars indicate + SD of three biological replicates. The asterisk indicates a significant difference by *t*-test analysis: ^∗∗^*P* < 0.01.

TABLE S1 Primers for quantitative real-time PCR (qPCR) and constructs.

TABLE S2 Important *cis*-elements found in the *VlPYL1* promoter using PLANT-CARE database analysis.

TABLE S3 The number of red spots 8 days after injection.

TABLE S4 The number of coloring fruits 16 days after injection.
