# Supplementary figures and images for: Characterization of the ABA Receptor VlPYL1 That Regulates Anthocyanin Accumulation in Grape Berry Skin
Source: Front Plant Sci. 2018 May 18;9:592. doi: 10.3389/fpls.2018.00592 (PMC5968127; doi:10.3389/fpls.2018.00592)

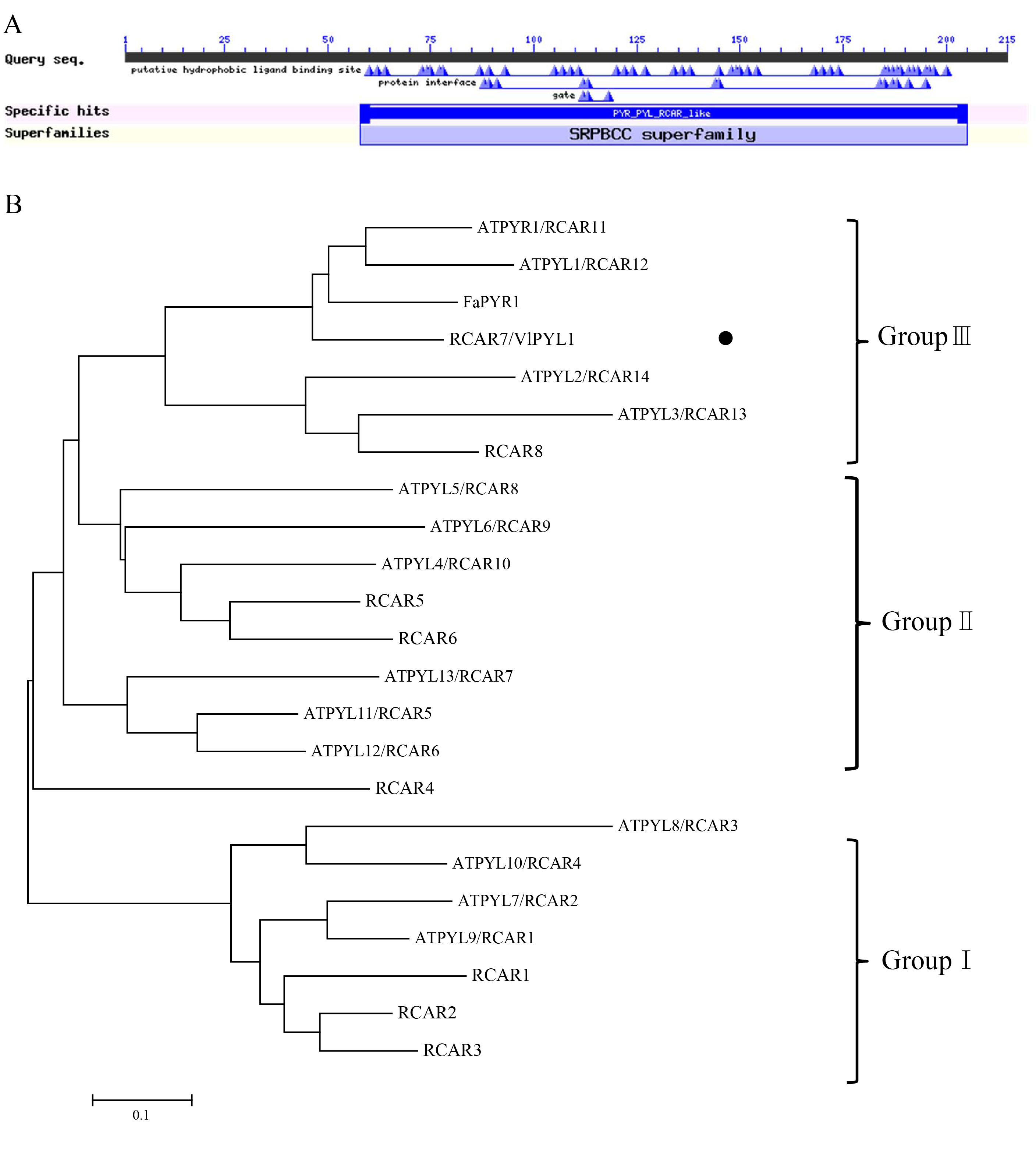

Supplement: FIGURE S1 — Sequence analysis of VlPYL1. (A) Finding conserved domains in the VlPYL1 protein. Conserved domains were determined for the VlPYL1 protein via a 215-amino acid polypeptide BLAST in the NCBI database (http://blast.ncbi.nlm.nih.gov/Blast.cgi). The PYR/PYL/RCAR-like family belongs to the SRPBCC (START/RHO-alpha-C/PITP/Bet-v1/CoxG/CalC) domain superfamily of proteins that binds hydrophobic ligands. SRPBCC domains have a deep hydrophobic ligand-binding pocket. (B) Phylogenetic tree showing the relationship between VlPYL1 and other PYR/PYL/RCAR proteins. The tree presented here is a Neighbor–Joining tree based on an amino acid sequence alignment. The numbers next to each node give bootstrap values for 1000 replicates. Genes and accession numbers are as follows: AtPYR1, At4g17870; At PYL1, At5g46790; At PYL2, At2g26040; At PYL3, At1g73000; At PYL4, At2g38310; At PYL5, At5g05440; At PYL6, At2g40330; At PYL7, At4g01026; At PYL8, At5g53160; At PYL9, At1g01360; At PYL10, At4g27920; At PYL11, At5g45860; At PYL12, At5g45870; At PYL13, At4g18620; FaPYR1, JF268669; RCAR1, VIT 215s0046g01050; RCAR2, VIT 216s0050g02620; RCAR3, VIT 202s0025g01340; RCAR4, VIT 210s0003g01335; RCAR5, VIT 208s0058g00470; RCAR6, VIT 213s0067g01940; VlPYL1/RCAR7, VIT 202s0012g01270 and RCAR8, VIT 204s0008g00890. [file Image_1.TIF]

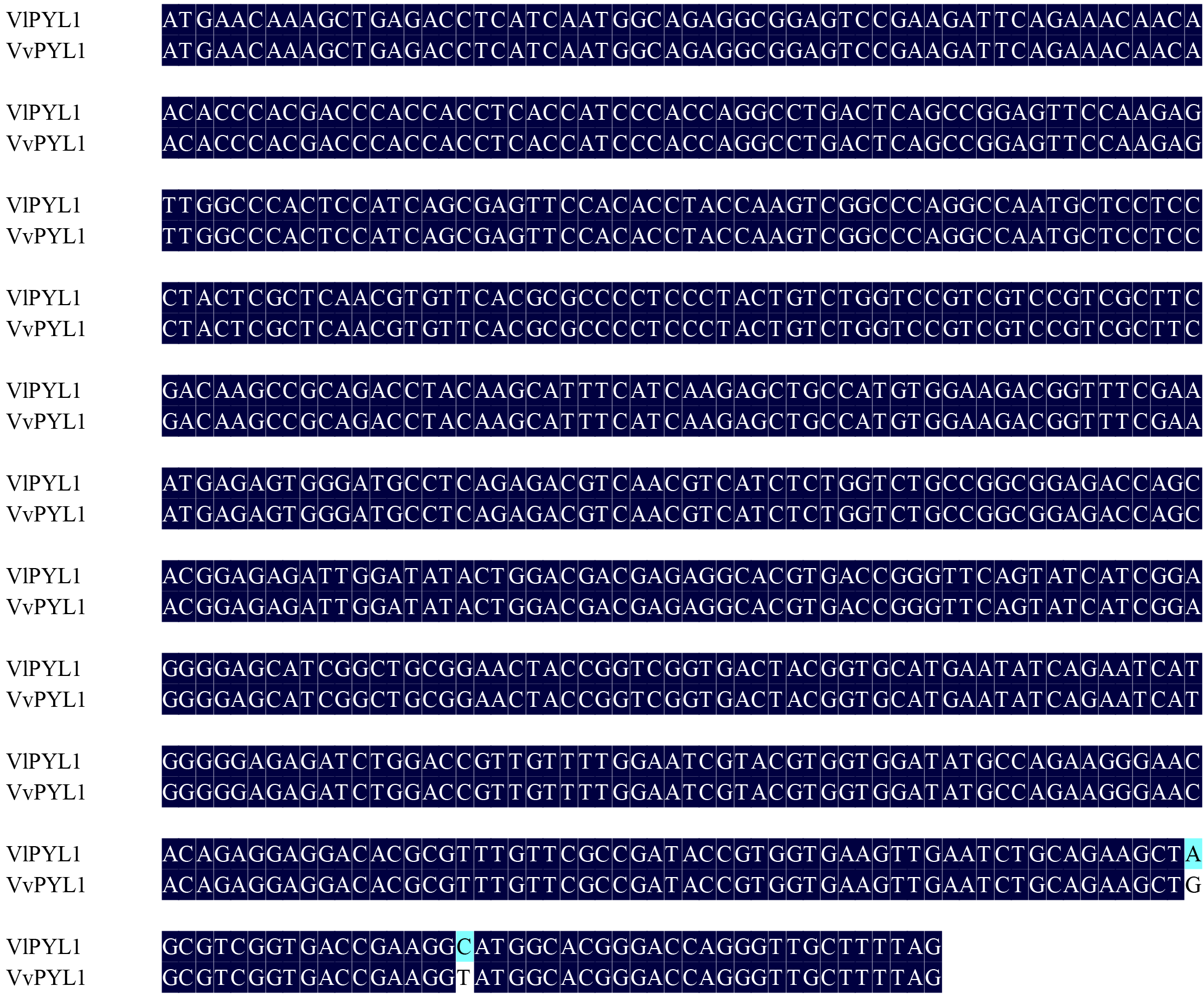

Supplement: FIGURE S2 — Coding-sequence analysis of VlPYL1 from “Kyoho” grape and VvPYL1 from Vitis vinifera cv. Muscat of Hamburg reported by Li et al. (2012). [file Image_2.TIF]

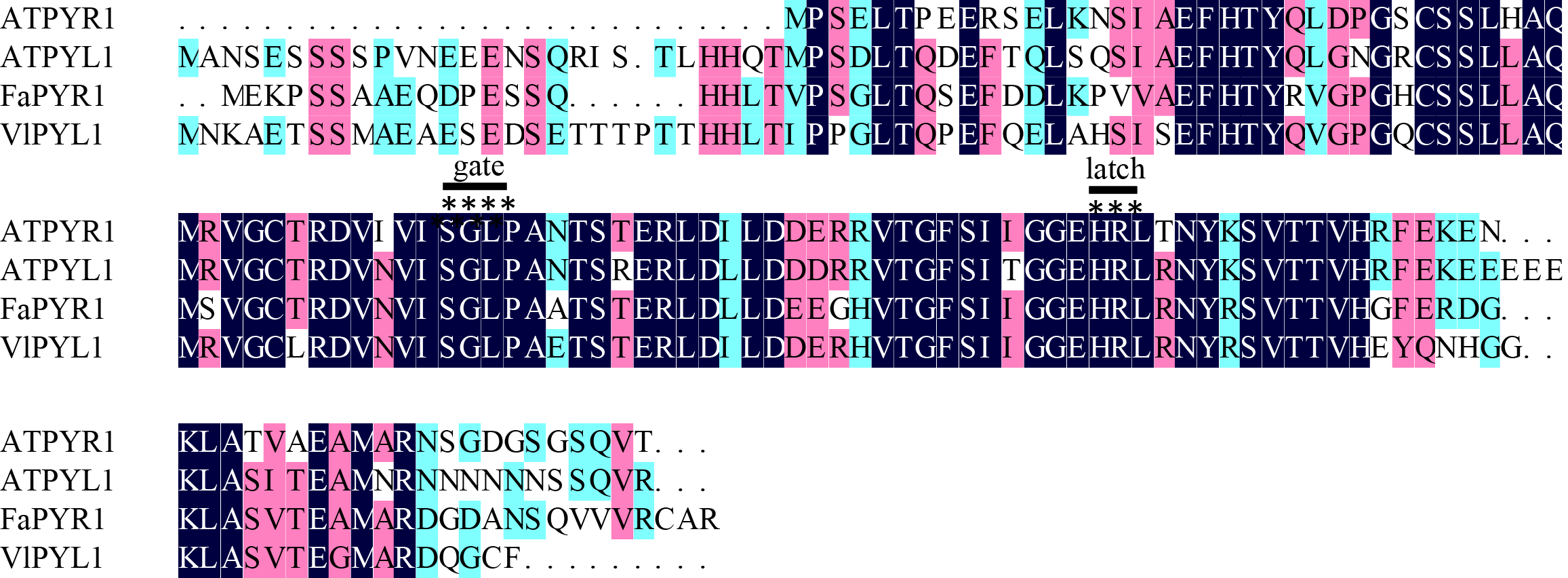

Supplement: FIGURE S3 — Amino acid sequence analysis of VlPYL1. Sequence alignment of the VlPYL1 protein with PYR1 and PYL1 in Arabidopsis and PYR1 in strawberries. Positions with identical amino acid residues are highlighted in blue, while similar amino acid residues are colored in pink and cyan. [file Image_3.TIF]

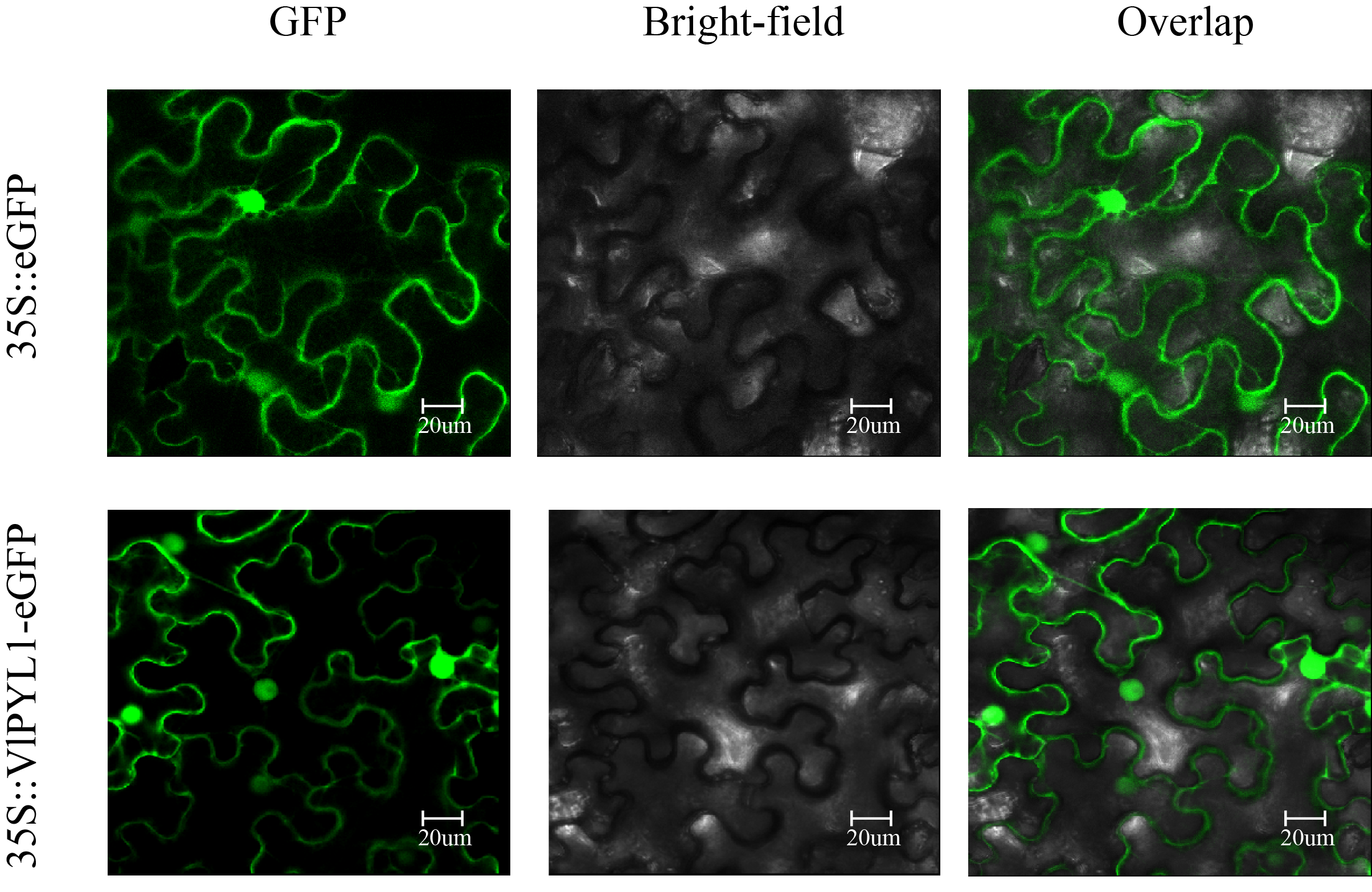

Supplement: FIGURE S4 — Subcelluar localization of VlPYL1 in tobacco leaves. Nicotiana benthamiana leaves were transiently infiltrated with A. tumefaciens GV3101 containing vectors expressing 2 × 35S::eGFP and 2 × 35S:: VlPYL1-eGFP. Confocal images were captured 72 h after inoculation. GFP image fluorescences are shown in the left panels, bright field images are shown in the middle panels, and overlap images are shown in the right panels. [file Image_4.TIF]

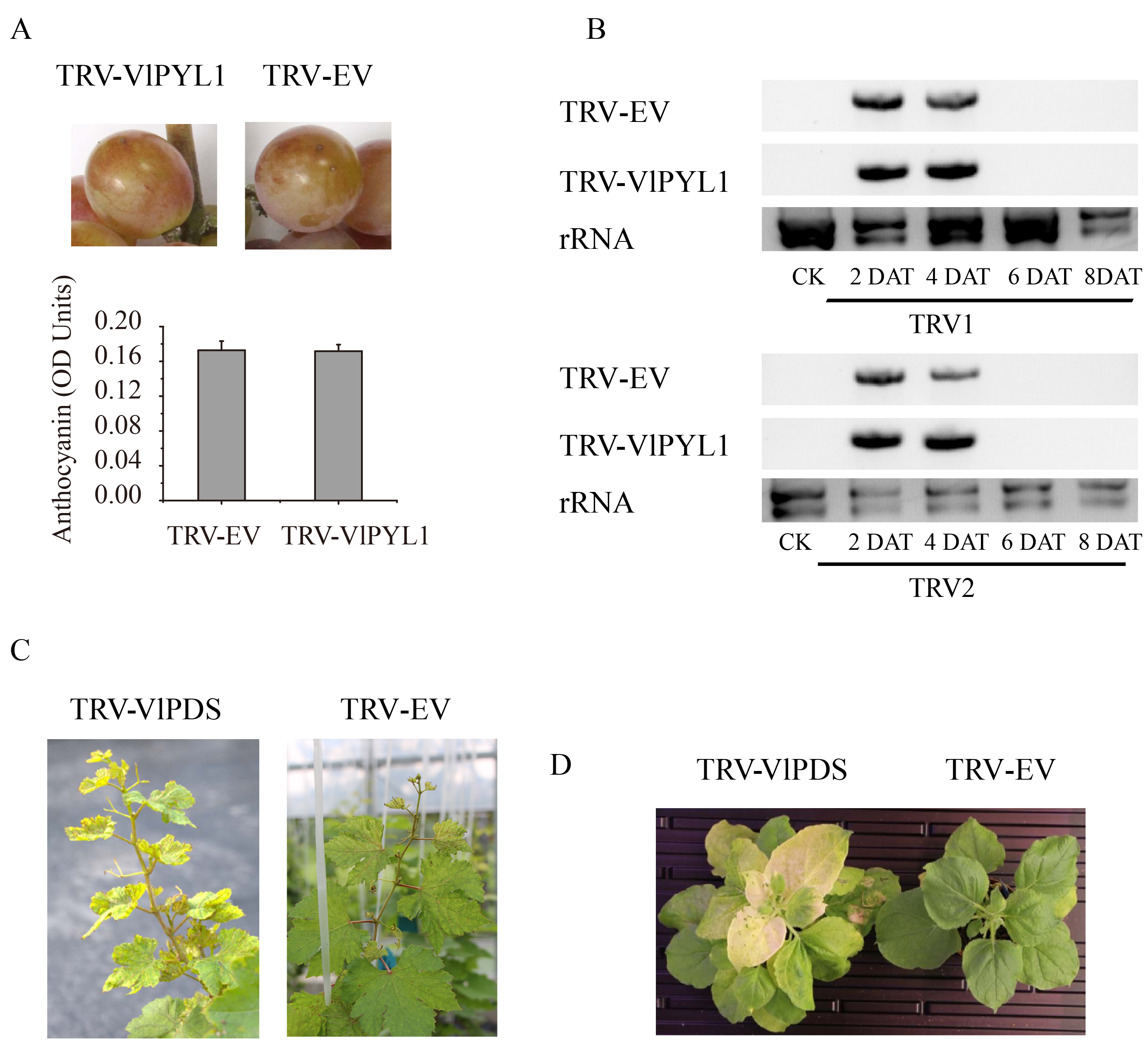

Supplement: FIGURE S7 — (A) Effect of TRV-VlPYL1 on grape fruit ripening. Note that both the control fruit and the RNAi fruit became red. The total anthocyanin content was measured in the control and TRV-RNAi fruits. (B) RT-PCR analysis of TRV expression in fruits. After infiltration, virus vector gene expression of both 647 bp pTRV1 and 367 bp pTRV2 was detected in fruits infiltrated with Agrobacterium containing TRV (lane 2 and lane 3: TRV-VlPYL1-treated grape fruits 2 and 4 days after infiltration) but were not detected in fruits infiltrated with Agrobacterium alone (lane 1) nor in TRV-VlPYL1-treated grape fruits 6 days (lane 4) and 8 days (lane 5) after infiltration. (C) TRV-VvPDS-treated grape plants 30 days after infiltration. (D) Mock-treated and TRV-VvPDS-treated tobacco plants 16 days after infiltration. The photobleached phenotype was observed in the plant (left) infiltrated with TRV-VvPDS, while the plant treated with TRV alone (right) remained green. [file Image_7.TIF]

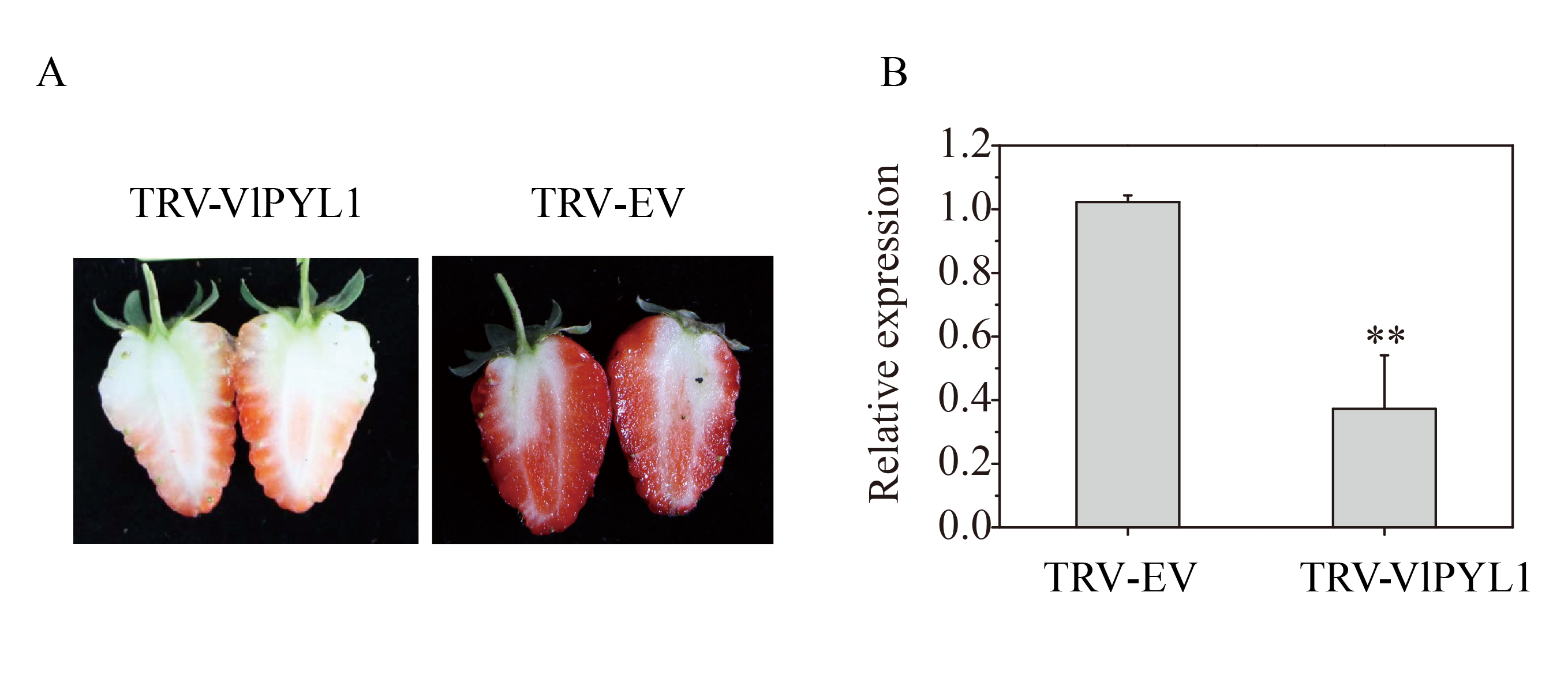

Supplement: FIGURE S8 — (A) Strawberry fruits agroinfiltrated with empty vector (EV) and agroinfiltrated fruit with VlPYL1-RNAi construct (RNAi) at 7 days after injection. (B) FaPYR1 expression by RT-qPCR of strawberry receptacle infiltrated with empty vector (Control) and VlPYL1-RNAi vector, 7 days after injection. Error bars indicate + SD of three biological replicates. The asterisk indicates a significant difference by t-test analysis: ∗∗P < 0.01. [file Image_8.TIF]
