# Supplementary material for: Characterization of the ABA Receptor VlPYL1 That Regulates Anthocyanin Accumulation in Grape Berry Skin
Source: Front Plant Sci. 2018 May 18;9:592. doi: 10.3389/fpls.2018.00592 (PMC5968127; doi:10.3389/fpls.2018.00592)
Supplement: TABLE S1 — Primers for quantitative real-time PCR (qPCR) and constructs. [file Table_1.DOC]

Supplementary Table S1. Primers for quantitative real-time PCR (qPCR) and constructs

| **Gene** | **Primer sequence 5’-3’** | **Gene ID** |
| --- | --- | --- |
| **a) For cloning** |  |  |
| ***VlPYL1-F*** | ATGAACAAAGCTGAGACCTCATCA | VIT_202s0012g01270 |
| ***VlPYL1-R*** | CTAAAAGCAACCCTGGTCCCG |  |
| ***VlPYL1-promoter-F*** | ATGGTCAACTTACGAATTTGGGTG | VIT_202s0012g01270 |
| ***VlPYL1-promoter-R*** | CGCCGCAAAAAGAGACGC |  |
| **b) For constructs** |  |  |
| ***pHB-VlPYL1-F*** | CGCAAGCTTATGAACAAAGCTGAGACCT | VIT_202s0012g01270 |
| ***pHB-VlPYL1-R*** | CGCGGATCCAAAGCAACCCTGGTCCCGT |  |
| ***TRV2-LIC-VlPYL1-F*** | CGACGACAAGACCCTGCGAGTTCCACAC  CTACCAAG | VIT_202s0012g01270 |
| ***TRV2-LIC-VlPYL1-R*** | GAGGAGAAGAGCCCTGCGTGTCCTCCTC  TGTGTTC |  |
| ***TRV2-LIC-VlPDS-F*** | CGACGACAAGACCCTCTCGTCCCAGTAA  ACCATTAGAGGTTG | VIT_209s0002g00100 |
| ***TRV2-LIC-VlPDS-R*** | GAGGAGAAGAGCCCTCCGGCCAAGTCA  GCATTTCATTATTC |  |
| **c) For detection vectors after injection^1^** |  |  |
| ***pHB-Hyg-F*** | TCGTTATGTTTATCGGCACTTTG | XM_003071606 |
| ***pHB-Hyg-R*** | GCGTCTGCTGCTCCATACAAG |  |
| ***TRV-RNA1-F*** | TTACAGGTTATTTGGGCTAG | AF406990 |
| ***TRV-RNA1-R*** | CCGGGTTCAATTCCTTATC |  |
| ***TRV-RNA2-F*** | TTACGACGAACCAAGGGAGTACTAC | AF406991 |
| ***TRV-RNA2-R*** | AGTCACAATTAGCCCTATTTAGATGT |  |
| **d) For qPCR** |  |  |
| ***AtACTIN2-F*** | CTTGCACCAAGCAGCATGAA | AT3G18780 |
| ***AtACTIN2-R*** | CCACCGATCCAGACACTGTACTT |  |
| ***AtRD29A-F*** | GTGCCGACGGGATTTGAC | AT5G52310 |
| ***AtRD29A-R*** | CTGATGCCTCACCGTATCCA |  |
| ***AtRD29B-F*** | GAAAGTGGCGGGAACTGTTG | AT5G52300 |
| ***AtRD29B-R*** | TCACCGTTGATCCTGTTTCTTTG |  |
| ***AtRAB18-F*** | TTCGGTCGTTGTATTGTGCTTT | AT5G66400 |
| ***AtRAB18-R*** | CCAGATGCTCATTACACACTCATG |  |
| ***AtKIN1-F*** | GGCAGCGGGAGGTGTTAAC | AT5G15960 |
| ***AtKIN1-R*** | TGACCCGAATCGCTACTTGTT |  |
| ***Vl-Actin-F*** | CTTGCATCCCTCAGCACCTT | VIT_204s0044g00580 |
| ***Vl-Actin-R*** | TCCTGTGGACAATGGATGGA |  |
| ***VlPYL1-F*** | GGTGACTACGGTGCATGAATATCA | VIT_202s0012g01270 |
| ***VlPYL1-R*** | CCAGCTTCTGCAGATTCAACTTC |  |
| ***VlNCED1-F*** | GGTGGTGAGCCTCTGTTCCT | VIT_219s0093g00550 |
| ***VlNCED1-R*** | CTGTAAATTCGTGGCGTTCACT |  |
| ***VlNCED2-F*** | AGTTCCATACGGGTTTCATGGG | VIT_210s0003g03750 |
| ***VlNCED2-R*** | CCATTTTCCAAATCCAGGGTGT |  |
| ***VlCYP707A1-F*** | GGTCACTTGGAGGGTAATTAC | VIT_203s0063g00380 |
| ***VlCYP1707A1-R*** | TGTTGTCGGCGATTTGATCCT |  |
| ***VlBG1-F*** | TGAACCTTACATAGTTGCCCACCAT | VIT_201s0011g00760 |
| ***VlBG1-R*** | AATCCCCATACATCAGAGGGTCAAT |  |
| ***VlBG2-F*** | ATAGTGAAGAAGAGGGCAGGCACG | VIT_207s0005g00380 |
| ***VlBG2-R*** | GCGGCCATATCTGCAAGAAAGTC |  |
| ***VlBG3-F*** | GCCGCAGAATAGTAGAAGACTTTGC | VIT_214s0006g01790 |
| ***VlBG3-R*** | GCAATATAAGGCTCGGTTGATGAGT |  |
| ***VlPP2C4-F*** | TGGGCTTTGGGATGTTATGT | VIT_211s0016g03180 |
| ***VlPP2C4-R*** | TGTGCAGGAGTCTCATCAGC |  |
| ***VlPP2C9-F*** | TTAAAGCCCTTCGTGAGCTG | VIT_206s0004g05460 |
| ***VlPP2C9-R*** | GACACCACGTCCCACAGAC |  |
| ***VlSnRK2.1-F*** | TTTTTGTGGCAAACCCAGAT | VIT_218s0001g06310 |
| ***VlSnRK2.1-R*** | CAGCTTCCTCCATCCATCAT |  |
| ***VlSnRK2.6-F*** | TGTTGGAACCCCTGCATACA | VIT_203s0063g01080 |
| ***VlSnRK2.6-R*** | CTGCAATCTTCCCGTCATATTCT |  |
| ***VlABF1-F*** | CACAGGATTGATGGGAAACC | VIT_218s0001g10450 |
| ***VlABF1-R*** | GAGCCTGCCCATTAACATTC |  |
| ***VlABF2-F*** | CAAGTGGATCTCCTGCCAATC | VIT_218s0001g10450 |
| ***VlABF2-R*** | GGGAACAGGTGAAACGGAAGA |  |
| ***VlMYBA1-F*** | TAGTCACCACTTCAAAAAGG | VIT_202s0033g00410 |
| ***VlMYBA1-R*** | GAATGTGTTTGGGGTTTATC |  |
| ***VlPAL-F*** | CATATCCACTGATGCAGAAG | VIT_200s2849g00010 |
| ***VlPAL-R*** | TCCCCTCACACATTGCAGTA |  |
| ***VlCHS-F*** | AAACTATGTGCTACAGTCC | VIT_214s0068g00920 |
| ***VlCHS-R*** | GACTACAGTTCAGAAATAA |  |
| ***VlCHI-F*** | CAGGCAACTCCAT TCTTTTC | VIT_213s0067g03820 |
| ***VlCHI-R*** | TTCTCTATGACTGCATTCCC |  |
| ***VlF3H-F*** | CCAATCATAGCAGACTGTCC | VIT_204s0023g03370 |
| ***VlF3H-R*** | TCAGAGGATACACGGTTGCC |  |
| ***VlDFR-F*** | GAAACCTGTAGATGGCAGGA | VIT_218s0001g12800 |
| ***VlDFR-R*** | GGCCAAATCAAACTACCAGA |  |
| ***VlUFGT-F*** | TAACACATTGTGGATGGAACTCAT | VIT_216s0039g02230 |
| ***VlUFGT-R*** | ACCTTCAATTCTCACTCCAATCTC |  |
| ***VlLDOX-F*** | AGGGAAGGGAAAACAAGTAG | VIT_202s0025g04720 |
| ***VlLDOX-R*** | ACTCTTTGGGGATTGACTGG |  |
| ***VlF3'H-F*** | ATTCGCCACCCTGAAATGAT | VIT_217s0000g07210 |
| ***VlF3'H-R*** | AGCCGTTGATCTCACAGCTC |  |
| ***VlFLS1-F*** | CAGGGCTTGCAGGTTTTTAG | VIT_218s0001g03470 |
| ***VlFLS1-R*** | GGGTCTTCTCCTTGTTCACG |  |
| ***VlANR-F*** | GCTGCTGTTACCATCAATCA | VIT_200s0361g00040 |
| ***VlANR-R*** | GCAGGATAGCCCCAAGTAGG |  |
| ***VlF3'5'H-F*** | GAAGTTCGACTGGTTATTAACAAAGAT | VIT_206s0009g02840 |
| ***VlF3'5'H-R*** | AGGAGGAGTGCTTTAATGTTGGTA |  |
| ***VlLAR1-F*** | AAATGAACTCGCATCTGTGT | VIT_201s0011g02960 |
| ***VlLAR1-R*** | CTGTGGGATGATGTTTTCTC |  |
