# Supplementary material for: Characterization of the ABA Receptor VlPYL1 That Regulates Anthocyanin Accumulation in Grape Berry Skin
Source: Front Plant Sci. 2018 May 18;9:592. doi: 10.3389/fpls.2018.00592 (PMC5968127; doi:10.3389/fpls.2018.00592)
Supplement: TABLE S2 — Important cis-elements found in the VlPYL1 promoter using PLANT-CARE database analysis. [file Table_2.DOC]

Supplementary Table S2. Important cis-elements found in *VlPYL1* promoter using PLANT-CARE database analysis

| **Cis-acting elements** | **Sequence** | **Copy number** | **Function** |
| --- | --- | --- | --- |
| **ABRE** | GCAACGTGTC, TACGTG | 2 | Involved in ABA responsiveness |
| **TC-rich repeats** | ATTCTCTAAC | 1 | Involved in defense and stress responsiveness |
| **LTR** | CCGAAA | 1 | Involved in low-temperature responsiveness |
| **ARE** | TGGTTT | 1 | Essential for the anaerobic induction |
| **W-box** | TTGAC**C** | 3 | Fungal elicitor responsive element |
| **ERE** | ATTTCAAA | 2 | Ethylene-responsive element |
| **TCA-element** | CAGAAAAGGA | 1 | Involved in salicylic acid responsiveness |
| **TGACG-motif** | TGACG | 2 | Involved in the MeJA-responsiveness |
