# Supplementary material for: Characterization of the ABA Receptor VlPYL1 That Regulates Anthocyanin Accumulation in Grape Berry Skin
Source: Front Plant Sci. 2018 May 18;9:592. doi: 10.3389/fpls.2018.00592 (PMC5968127; doi:10.3389/fpls.2018.00592)
Supplement: TABLE S3 — The number of red spots 8 days after injection. [file Table_3.DOC]

Supplementary Table S3. The number of red spots in 8 d after injection

|  | **Number of red spots 8 d after treatment** | **Total number** |
| --- | --- | --- |
| **Empty vector-OE** | 7 | 50 |
| ***VlPYL1*-OE** | 16 | 50 |
| **Empty vector-TRV** | 6 | 50 |
| ***VlPYL1*-RNAi** | 8 | 50 |
