# Supplementary material for: Characterization of the ABA Receptor VlPYL1 That Regulates Anthocyanin Accumulation in Grape Berry Skin
Source: Front Plant Sci. 2018 May 18;9:592. doi: 10.3389/fpls.2018.00592 (PMC5968127; doi:10.3389/fpls.2018.00592)
Supplement: TABLE S4 — The number of coloring fruits 16 days after injection. [file Table_4.DOC]

Supplementary Table S4. The number of colouring fruits in 16 d after injection

|  | **Number of coloured fruits 16d after treatment** | **Number of decayed fruits** | **Total number** |
| --- | --- | --- | --- |
| **Empty vector-OE** | 40 | 10 | 50 |
| ***VlPYL1*-OE** | 41 | 9 | 50 |
| **Empty vector-TRV** | 36 | 14 | 50 |
| ***VlPYL1*-RNAi** | 35 | 15 | 50 |
